# N^6^-methyladenine is an epigenetic marker of mammalian early life stress

**DOI:** 10.1038/s41598-017-18414-7

**Published:** 2017-12-22

**Authors:** Stacey L. Kigar, Liza Chang, Candace R. Guerrero, Jacqueline R. Sehring, Amelia Cuarenta, Laurie L. Parker, Vaishali P. Bakshi, Anthony P. Auger

**Affiliations:** 1 Molecular and Cellular Pharmacology Training Program, University of Wisconsin-Madison, Madison, WI 53706 United States; 2 Department of Psychology, University of Wisconsin-Madison, University of Wisconsin-Madison, Madison, WI 53706 United States; 3 Department of Biochemistry, Molecular Biology, and Biophysics, University of Minnesota, Minneapolis, MN 55455 United States; 4 Department of Psychiatry, University of Wisconsin-Madison, Madison, WI 53706 United States; 5 Neuroscience Training Program, University of Wisconsin-Madison, Madison, WI 53706 United States

## Abstract

Recent evidence described 6-methyladenine (6 mA) as a novel epigenetic regulator in a variety of multicellular species, including rodents; however, its capacity to influence gene expression in the mammalian brain remains unknown. We examined if 6 mA is present and regulated by early life stress associated with predator odor exposure (POE) within the developing rat amygdala. Our results provide evidence that 6 mA is present in the mammalian brain, is altered within the *Htr2a* gene promoter by early life stress and biological sex, and increased 6 mA is associated with gene repression. These data suggest that methylation of adenosine within mammalian DNA may be used as an additional epigenetic biomarker for investigating the development of stress-induced neuropathology.

## Introduction

There is considerable evidence suggesting that precise regulation of 5-methylcytosine (5 mC) during brain maturation is necessary to achieve species-typical behaviors later in life, and that early life experiences can alter epigenetic programming^1–3^. Of particular clinical interest, early life stress (ELS) can negatively affect one’s wellbeing into adulthood, potently regulating the epigenome (for review, see^4–6^) and correlates with increased diagnosis of affective disorders such as anxiety and post-traumatic stress disorder (PTSD)^7–10^. The rate of diagnosis for these and other psychiatric conditions varies greatly between males and females^11^, thus a greater understanding of how biological sex confers risk or resilience during organismal development is critically important for improved treatment strategies.

We and others have previously described sex differences in the neuronal epigenome with respect to 5-methylcytosine (5 mC)^12,13^. Very recently, the presence of 6-methyladenine (6 mA) and its manipulation by the enzyme Alkbh1 was characterized biochemically in mouse embryonic stem cells^14^. This finding builds on a series of 2015 publications demonstrating the functional existence of 6 mA in a variety of multi-cellular organisms —including *Chlamydomonas* ^15^, *C*. *elegans* ^16^, and *Drosophila* ^17^—and other work showing that 6 mA is present in mammalian cell lines at very low levels of abundance^18^. These discoveries herald a major paradigm shift, as 6 mA was previously thought to exist exclusively in bacteria and some unicellular organisms^19^. Given the environmentally-sensitive nature of this modification in bacteria^20^, and data suggesting that 6 mA is enriched in adult brain tissue of *Drosophila* ^17^, we sought to determine whether 6 mA could be found in the mammalian brain and whether it was responsive to the neonatal environment.

**Figure 1:**
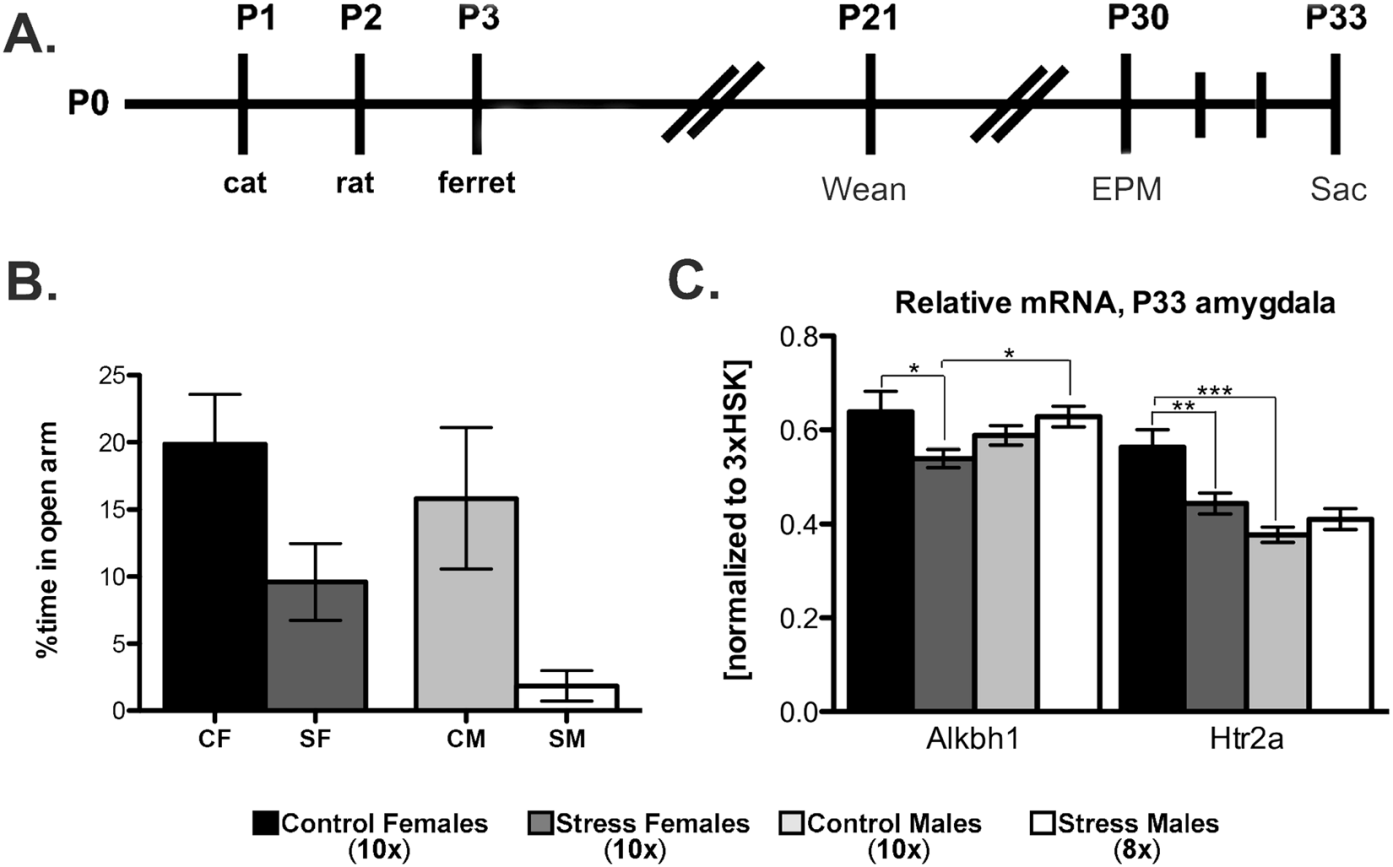
Neonatal predator odor exposure (POE) causes lasting effects on juvenile behavior and gene expression in the amygdala. (**A**) Mixed-sex litters were acutely exposed to variable predator odor for three consecutive days. Half of the animals were sacrificed for gene expression analysis 30 m post-exposure on P3; the remaining half was raised undisturbed before behavior testing and sacrifice at P33. (**B**) The elevated plus maze (EPM) shows lasting effect of neonatal, POE stress on anxiety-like behavior. (**C**) RT-qPCR analysis of the juvenile amygdala reveals significant difference in the mRNA expression of the 6 mA demethylase Alkbh1 and Htr2a. Values shown as mean ± SEM. ***p < 0.001, **p < 0.01, *p < 0.05.

We developed a predator odor exposure (POE) stress paradigm for neonates to model the lasting effects of early life adversity in the adolescent brain (Fig. 1A), adapted from well-established methods in the lab of Dr. Bakshi and others^21–27^. We chose to vary the predator odors to eliminate habituation as a potential confound, and checked daily weight gain and for the presence of milk in the stomach to ensure maternal feeding behavior wasn’t disrupted. We examined the impact of predator odor exposure within the developing amygdala, as this area receives sensory information from both olfactory and pheromonal cues, and furthermore has a modulatory role in fear acquisition and anxiety^28^. We focused on amygdalar mRNA expression of 5-hydroxytryptamine (5-HT, or serotonin) receptors Htr2a and Htr1a, which are well-known for mediating anxiety-like behavior^29,30^. We then examined *Htr2a* and *Htr1a* promoter methylation and chromatin status to assess whether predator odor-induced ELS altered 6 mA levels within the developing amygdala. Herein we report not only the presence of 6 mA within the developing mammalian brain, and amygdala specifically, but also a sex difference in 6 mA expression levels associated with gene repression at the Htr2a promoter. Importantly, we find that ELS during the early neonatal period results in lasting, sex-specific changes in 6 mA levels and anxiety-like behavior.

## Results

### POE induces anxiety-like behavior

We used the elevated plus maze (EPM) to examine anxiety-like behavior; POE-stress reduced time spent in the open arm for both males and females (two-way ANOVA; *F*_(1,34)_ = 10.337, *p* = 0.003; Fig. 1B). The number of entries into the open arm was similarly decreased in stressed animals (two-way ANOVA; *F*_(1,34)_ = 9.58, *p* = 0.004; Supplementary Figure 1A), though incidences of neither stretch-attend posture, head dips, or overall locomotor activity were altered due to stress (Supplementary Figure 1B–D).

### POE results in temporal- and sex-specific changes to mRNA expression of the 6 mA demethylase Alkbh1 and serotonin receptors

Recent reports suggest that *alkB* homologue 1 (Alkbh1) is a mammalian 6 mA demethylase^14^. We observed an interaction of stress and biological sex on Alkbh1 mRNA expression at postnatal day 33 (P33) in the juvenile amygdala (two-way ANOVA; *F*_(1,36)_ = 5.938, *p* = 0.02) (Fig. 1C). Post hoc analysis indicated stressed males express higher levels of Alkbh1 mRNA than stressed females (Tukey’s *post hoc*; *q* = 3.061, *p* = 0.037); furthermore, stressed females expressed lower levels of Alkbh1 mRNA when compared to control females (Tukey’s *post hoc*; *q* = 3.415, *p* = 0.021). This would imply that control females should have less 6 mA compared to stressed females as a result of Alkbh1-mediated methylation removal, though the temporal and regional specificity of Alkbh1 as an epigenetic regulator remains to be determined.

We next looked at the serotonergic system given its known role in mediating anxiety-like behaviors. At P33, there was a main effect of sex on the expression of Htr2a (two-way ANOVA; *F*_(1,36)_ = 18.896, *p* < 0.001) and an interaction between stress and sex (two-way ANOVA; *F*_(1,36)_ = 9.116, *p* = 0.005) (Fig. 1C). Post hoc analysis revealed a sex difference, where control males had less Htr2a mRNA expression than females, and a stress effect in females where Htr2a mRNA expression was suppressed and more similar to male-typical levels [(Tukey’s *post hoc*; *q* = 7.269, *p* < 0.001); treatment effect in females (Tukey’s *post hoc*; *q* = 4.642, *p* = 0.002)]. We also examined Htr1a mRNA expression at P33 and found an interaction effect (group by sex; two-way ANOVA; *F*_(1,35)_ = 4.207, *p* = 0.048) (Supplementary Figure 2A). In this case, post hoc analysis revealed a sex difference in the opposite direction of Htr2a mRNA; specifically, control males had higher levels of Htr1a mRNA (Tukey’s *post hoc*; *q* = 3.954, *p* = 0.009), and stressed females had higher, more male-like levels of mRNA compared to control females (Tukey’s *post hoc*; *q* = 3.412, *p* = 0.022).

Neonatally, there was a main effect of sex on Htr2a expression at P3, where females expressed higher levels than males (two-way ANOVA; *F*_(1,21)_ = 7.773, *p* = 0.011) (Supplementary Figure 2B). Furthermore, a main effect of stress revealed increased Htr2a expression (treatment; two-way ANOVA; *F*_(1,21)_ = 10.124, *p* = 0.005). Htr1a mRNA expression was unaltered by sex or stress at this time point.

### Impact of stress on adenine methylation in the Htr2a promoter

**Figure 2:**
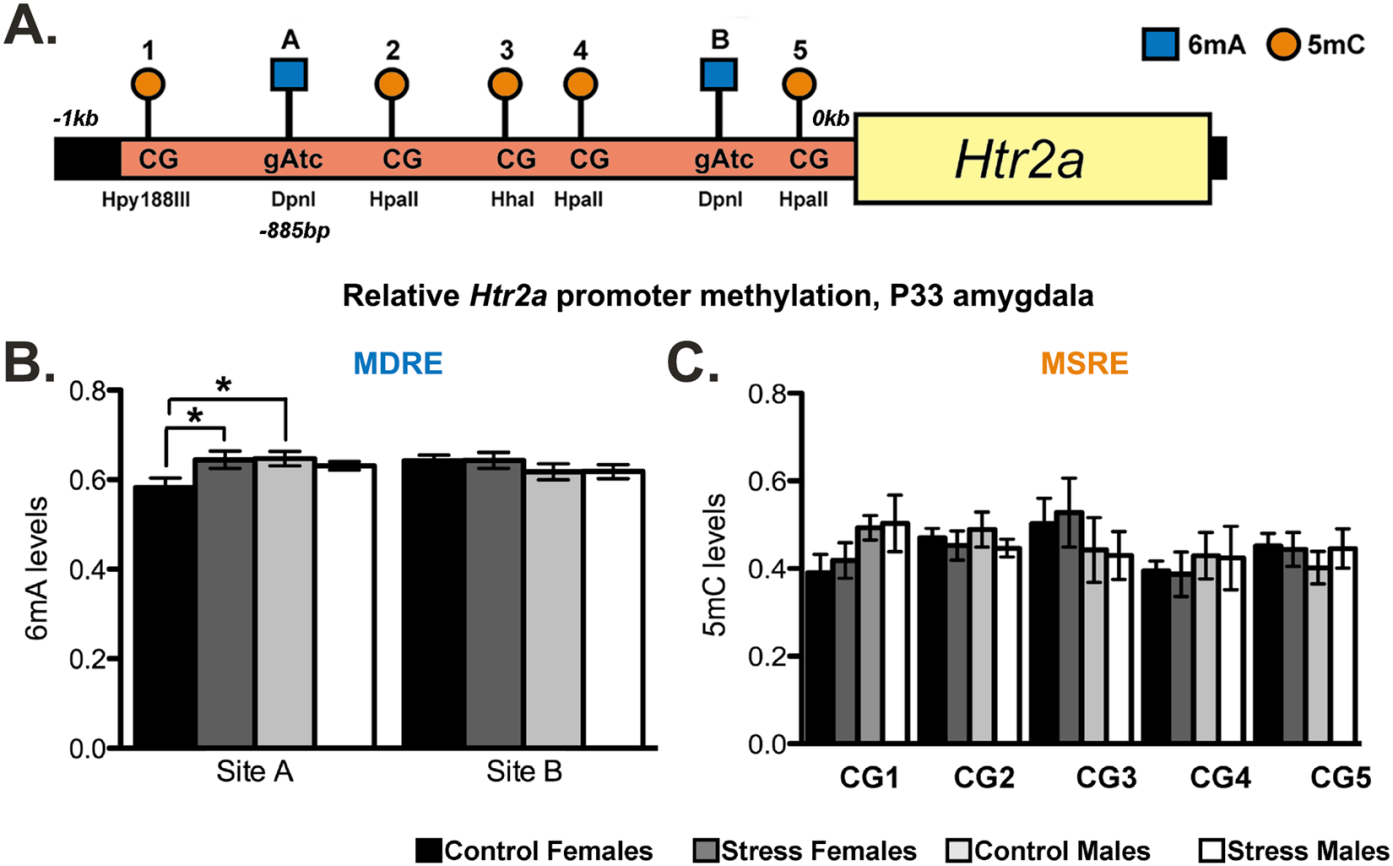
Methylation dependent and sensitive restriction enzyme digests reveal sex and stress effects on 6 mA levels. (**A**) Schematic of Htr2a gene promoter, showing individual methylation sites analyzed by restriction enzyme digestion of DNA coupled with qPCR. (**B**) MDRE analysis shows modest but significant sex and treatment effects on global amygdalar 6 mA abundance at Site A within the Htr2a promoter. (**C**) No significant differences in 5 mC levels were observed via MSRE in this region of the Htr2a promoter. Values shown as mean ± SEM, *p < 0.05.

To analyze global, amygdalar DNA content, we used a methylation-dependent restriction enzyme (MDRE) assay followed by quantitative PCR to examine the two GmATC DpnI cut sites, arbitrarily referred to as A and B, found in the neuron-specific region of the *Htr2a* promoter^31^. At P33, both stressed and control males showed higher levels of 6 mA than control females, and stressed females had more male-typical levels (Fig. 2B). At Site A, an interaction was observed (stress by sex; two-way ANOVA; *F*_(1,34)_ = 4.579, *p* = 0.04). Post hoc analysis revealed a sex difference between control males and females, where males had higher levels of methylation (Tukey’s *post hoc*; *q* = 3.696, *p* = 0.013), and also a stress effect in females, where stressed females had higher levels of methylation (Tukey’s *post hoc*; *q* = 3.545, *p* = 0.017). Correlation between 6 mA levels at Site A and Htr2a mRNA levels closely approached significance (*p* = 0.0509; Supplementary Figure 3). No significant differences were observed at Site B.

At P3, sex differences in relative 6 mA levels were also observed, where males had higher levels than females, at Site A (two-way ANOVA; *F*_(1,20)_ = 7.288, *p* = 0.015). At Site B, we also observed a main effect of sex, where males had greater levels of 6 mA (two-way ANOVA; *F*_(1,20)_ = 6.094, *p* = 0.023), though there was no main effect of stress (Supplementary Figure 2C).

### Impact of stress on cytosine methylation in the Htr2a promoter

Again looking at global amygdalar DNA, we next used a variety of methylation-sensitive restriction enzymes (MSREs) to investigate CpG methylation sites within this region of the *Htr2a* promoter. We found no interactions or main effects for 5 mC levels in the *Htr2a* promoter at either P33 (Fig. 2B) or P3 (Supplementary Figure 2D). At this time, we cannot exclude the possibility of altered 5 mC levels in either subnuclei of the amygdala or within specific cell subtypes. We also cannot exclude alterations in 5 mC at sites for which MSREs do not exist.

### Detection of 6 mA in the P35 amygdala and sex differences in total 5 mC

**Figure 3:**
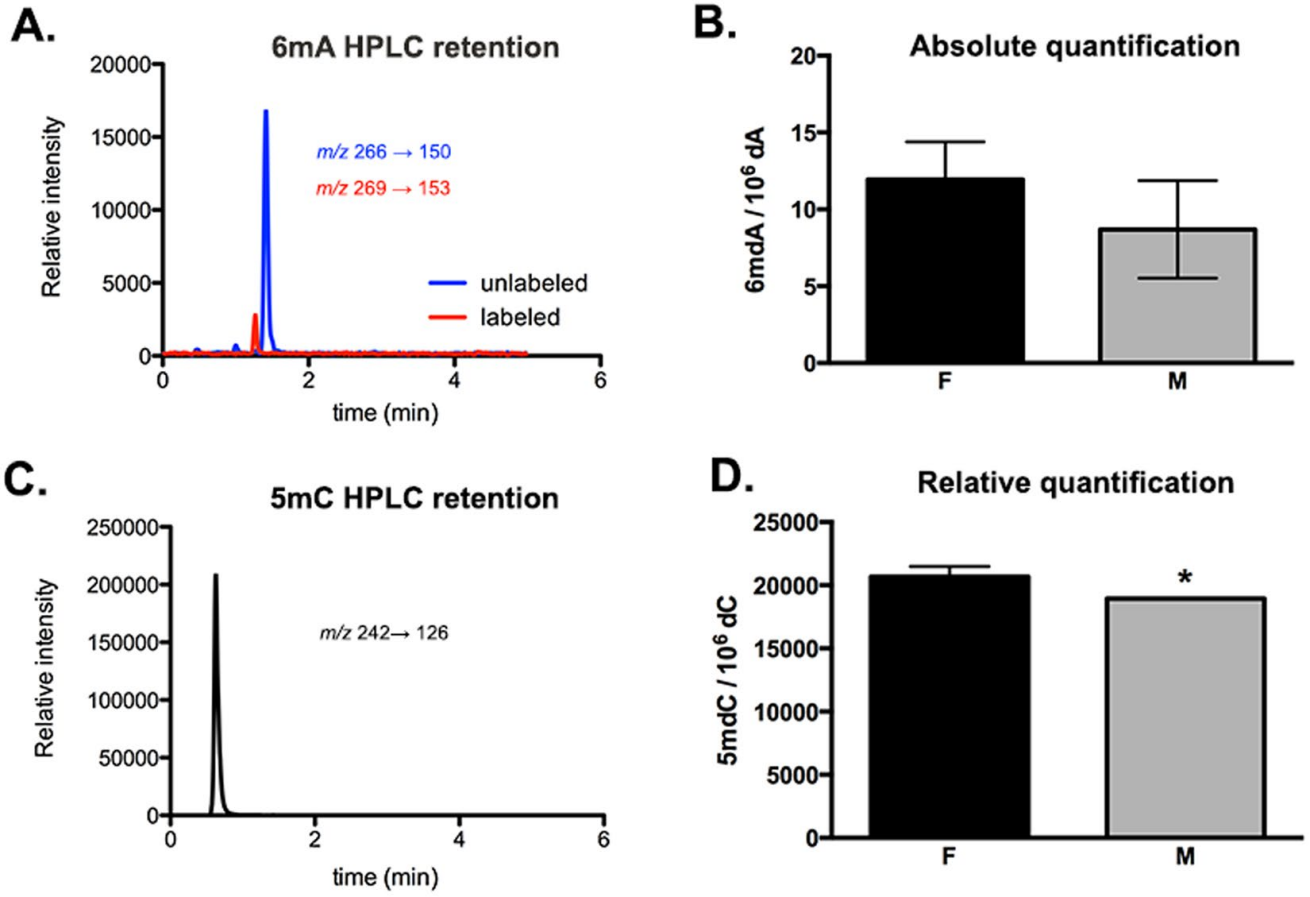
6 mA and 5mC abundance in the juvenile amygdala of unhandled male and female rats at P35 determined by mass spectrometry. (**A**) Selected-ion chromatogram (SICs) for monitoring the MRM transitions for unlabeled and labeled 6 mA. (**B**) Absolute levels of 6 mA isolated from amygdala tissue in female and male rats. (**C**) SIC for monitoring the transition of 5 mC. (**D**) Sex differences in the relative levels of 5 mC isolated from amygdala tissue. Values shown as mean ± SEM. *p < 0.05.

To further investigate these observations, we turned to an age-matched group of non-handled males and females aged P35 given the relative ease of obtaining sufficient quantities of genomic DNA. With an isotope labeled, spiked 6 mA internal standard for mass spectrometry analysis, we quantified the overall abundance of 6 mA in the P35 rat amygdala to be approximately 0.13% of the total DNA population, or approximately ten 6 mA adducts per every 10^6^ adenines (Fig. 3B). This number is consistent with recent results in mouse embryonic stem cells^14^. No sex difference was observed in the global levels of genomic 6 mA, which we note does not conflict with location-specific sex differences in the epigenome.

By comparison, 5 mC represented approximately 0.45% of the total DNA population, or ~2% of all cytosines (Fig. 3D); this number is in good agreement with previously published levels of the 5 mC adduct in rat brain^32^. A modest but significant ~10% sex difference in overall levels of 5 mC was observed (*t* test; *t*(5) = 3.617, *p* = 0.0153), where females had more 5 mC than males.

### Confirmation of Htr2a methylation findings via MeDIP analysis

**Figure 4:**
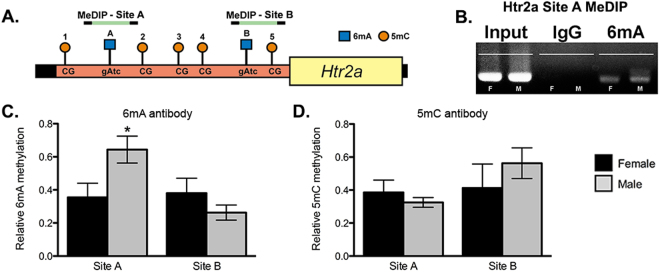
Methylated DNA immunoprecipitation (MeDIP) analysis of the Htr2a promoter confirms site-specific sex-differences in relative levels of 6 mA. (**A**) Schematic of Htr2a gene promoter, showing areas investigated by qPCR after either 6 mA or 5 mC MeDIP. (**B**) Representative DNA gel for 6 mA MeDIP of Htr2a promoter DNA at Site A; a positive input control and negative IgG control for male and female samples are shown for comparison. (**C**) Males have higher levels of 6 mA at Site A, but not Site B, in the Htr2a promoter within the P35 amygdala. (**D**) No significant differences found using 5 mC MeDIP. Values shown as mean ± SEM. *p < 0.05.

We next used these same genomic DNA samples for both 6 mA and 5 mC MeDIP analysis. The 6 mA antibody has been well-characterized biochemically elsewhere^17^ and we confirmed specificity of the assay using RNAse and DNAse treatments (data not shown). We found sex differences in 6 mA abundance that matched those found with the MDRE assay described above [Site A: (*t* test; *t*(9) = 2.438, *p* = 0.0375); Site B: (*t* test; *t*(7) = 1.321, *p* = 0.2280)] (Fig. 4B,C). There is one CpG site contained within the Site A primer set; however, no sex difference was observed at this location using the 5 mC antibody (*t* test; *t*(9) = 0.8075, *p* = 0.4402). There were also no sex differences in 5 mC levels using primers for Site B (*t* test; *t*(8) = 0.8704, *p* = 0.4094) (Fig. 4D).

### Methylation analysis of the Htr1a promoter

We observed no long-term methylation changes in either 6 mA (Supplementary Figure 4B) or 5 mC (Supplementary Figure 4C) using enzyme-based methods in POE-treated animals. For confirmation, we used a MeDIP assay in non-handled, P35 males and females as before. Again, no sex differences were observed in either 6 mA or 5 mC (data not shown). This suggests that 6 mA modifications by the environment are gene-specific.

### Effects on chromatin and Sp1 transcription factor binding

**Figure 5:**
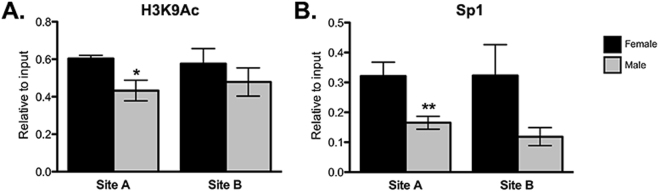
Chromatin immunoprecipitation (ChIP) in unhandled P35 male and female amygdalae to examine transcriptional activation of Sites A and B within the Htr2a promoter. (**A**) Females show significantly greater levels of H3K9 acetylation at Site A, but not at Site B. (**B**) Females show significantly greater binding of the transcription factor Sp1 at Site A, and trend towards greater binding at Site B. Values shown as mean ± SEM. **p < 0.01, *p < 0.05.

To examine whether N^6^-methyladenine was associated with a less-active chromatin state, we used a second cohort of non-handled P35 males and females for ChIP and probed for histone acetylation on core histone protein H3 at lysine 9 (H3K9), as this is a well-known marker of active gene expression^33^ (Fig. 5A). We observed a sex difference in H3K9 acetylation at Site A in the *Htr2a* promoter, where females had more H3K9 acetylation (*t* test; *t*(8) = 2.436, *p* = 0.0408). There was no sex difference at Site B (*t* test; *t*(9) = 0.8783, *p* = 0.4026).

Sp1 has previously been characterized as a transcription factor important for Htr2a mRNA transcription^34^. We observed a sex difference in Sp1 pull-down at Site A, but not Site B, with females having more Sp1 pull-down [Site A: (*t* test; *t*(9) = 3.263, *p* = 0.0098); Site B: (*t* test; *t*(8) = 1.899, *p* = 0.0941)] (Fig. 5B).

### Examination of Line1 and Bex4

The recent identification of 6 mA in the invertebrate genome found vast enrichment in *Drosophila* retrotransposon elements. We thus examined the presence of 6 mA in an abundant retrotransposon of the mammalian genome, long interspersed nuclear element 1 (Line1). Although 6 mA was highly enriched in an untranslated region of the Line1 promoter (Supplementary Figure 5), we saw no significant difference in the expression of Line1 mRNA in the early life stress samples (Supplementary Figure 2A).

A recent report from Wu, *et al*. found that 6 mA was highly enriched on the X-chromosome in embryonic stem cells^14^. We examined mRNA expression of brain expressed X-linked 4 (Bex4), a candidate from their array data that is expressed in the brain and found no significant differences by either treatment or sex (Supplementary Figure 2A).

## Discussion

The major contribution of this paper is our demonstration that within a discrete location of the genome, sexually dimorphic 6 mA levels are both present and altered by predator odor exposure (POE), thus making 6 mA an epigenetic marker of early life stress. These data support the novel conclusion that environmental stressors during neonatal development result in sex-specific changes to 6 mA levels in the adolescent mammalian brain. To test this, we used variable POE in neonatal rats to examine the long-term impact of early life stress (ELS) via predator odor-induced fear on anxiety-like behavior. Predator odor is thought to be a potent, innate, and ecologically relevant stressor, and works effectively in both sexes and across developmental time points. As with other models of early life stress, i.e. maternal separation (MS), we find that neonatal POE causes a lasting effect on juvenile anxiety-like behavior^35^, significantly decreasing time spent in the open arm of the EPM for both males and females (Fig. 1B).

The amygdala responds robustly to POE across development^21,36^, and plays a well-characterized role in fear learning and anxious behaviors^37^. We therefore collected amygdala tissue at two time points—immediately following stress exposure at P3, and after EPM testing at P33—to investigate sex-specific changes across development in gene expression due to ELS. We looked at Htr2a, one of several serotonin receptors that has been extensively studied due to its role in mediating anxiety-like behaviors; we found increased mRNA expression 30 minutes post-predator odor stress in both the neonatal male and female amygdala (Supplementary Figure 2B), perhaps indicative of heightened transcriptional activation. We also observed higher Htr2a mRNA expression in control females compared to control males at both time points. In the juvenile period, Htr2a mRNA expression in females exposed to POE was decreased relative to control females, but further decreases in stressed males’ expression was not observed (Fig. 1C), suggesting that lasting, epigenetic programming of amygdalar Htr2a expression due to stress is sex-specific. This is consistent with previously published data from our lab in which we observed that Htr2a expression is influenced by both biological sex and the neonatal environment in juveniles^38^.

To determine if Htr2a expression is related to N^6^-adenine methylation, we examined a region in the *Htr2a* gene promoter that is thought to be important for neuronal expression^31^ (Fig. 2A). We first exploited DamI methylation-dependent restriction enzyme (MDRE) cutting activity in tandem with qPCR amplification^39^, which allowed us to examine the occurrence of 6 mA at specific sites within the heterogeneous cell population of the amygdala. We reasoned that selective examination of specific sites using highly-sensitive, qPCR-based methods may reveal ELS-induced changes to 6 mA despite its low abundance in the genome^18^. Indeed, at a well-conserved area of the promoter^40^, we observed both a sex difference and a juvenile female-specific stress effect on 6 mA methylation (Fig. 2B) that may correlate with Htr2a mRNA expression (Supplementary Figure 3). An overall sex difference in 6 mA was present at both DamI sites in the *Htr2a* promoter neonatally (Supplementary Figure 2C), but was not seen proximal to the transcriptional start site (TSS) in juveniles (Fig. 2B). At both time points, the sex-difference in 6 mA methylation was consistent with a repressive effect on mRNA expression in males; additionally, the ELS-induced increase in 6 mA levels was consistent with a repressive effect on Htr2a mRNA levels in the female juvenile brain (Fig. 1C). These data suggest that N^6^-adenine methylation levels within the developing amygdala are sensitive to both biological sex and ELS. We observed no significant differences in 5 mC using a similar method at either time point (Fig. 2C, Supplementary Figure 2D), though we cannot rule out either cell-type or regionally-specific changes in 5 mC methylation. We furthermore can’t rule out 5 mC-mediated changes to Htr2a mRNA expression in other regions of the promoter. However, this global view of the amygdalar genome may suggest that adenine methylation exerts relatively greater influence over Htr2a gene expression within this region. No effects of 6 mA or 5 mC methylation were observed within the *Htr1a* promoter examined (Supplementary Figure 5), suggesting that alterations to adenine methylation levels may be specific to certain gene promoters or regions.

Resurgent interest in 6 mA as an epigenetic modifier in eukaryotes prompted the discovery of enzymes responsible for removing this mark on DNA. While no one has yet characterized a eukaryotic *de novo* deoxyadenosine methylase, DNA 6 mA demethylase (DMAD) in *Drosophila* ^17^ and Alkbh1 in mice^14^ have both been rigorously tested and shown to be 6 mA DNA demethylases that alter transcription of RNA. Alkbh1 appears to function analogously to Tet1-mediated demethylation of 5 mC. In Alkbh1 knockout mouse embryonic stem cells, levels of 6 mA were elevated, which correlated with gene repression, corroborated recently^41^. This effect is opposite to that seen with DNA 6 mA demethylase (DMAD) in *Drosophila* ^17^; DMAD knockout raised 6 mA levels and *increased* gene expression. This suggests that while 6 mA is associated with increased gene expression in *Drosophilia*, in rodents it may be associated with gene repression—a conclusion supported by the data presented herein. To our knowledge, studies examining environmental effects on Alkbh1 mRNA expression have not been conducted. At P33, we found an interaction between biological sex and POE in which stressed females expressed significantly less Alkbh1 (Fig. 1C), which should concomitantly increase levels of 6 mA. Therefore, we used siRNA-knockdown of Alkbh1 in murine cell culture to determine whether there were changes in 6 mA methylation at either Site A or Site B within the mouse *Htr2a* promoter; however, we observed no changes in 6 mA levels (data not shown). Given a dearth of information about the factors responsible for manipulating 6 mA within species-, temporal-, and tissue-specific genetic contexts, at present we cannot conclude whether Alkbh1 acts analogously in the developing rat amygdala and within the *Htr2a* promoter. Therefore, future studies are needed to determine the factors involved in 6 mA regulation within the developing brain.

To further verify the presence of 6 mA levels within the mammalian brain, we used two additional techniques, mass spectrometry and methylated DNA immunoprecipitation (MeDIP), in a cohort of non-handled male and female rat amygdalae collected at P35. We replicated the sex difference in Fig. 2B, observing increased 6 mA levels at Site A of the *Htr2a* promoter in males (Fig. 4C), and found no significant differences in *Htr2a* 5 mC methylation (Fig. 4D) or *Htr1a* methylation (data not shown), consistent with the MDRE assay (Fig. 2C). Given the robust presence of 6 mA in *Drosophila* retrotransposons^17^, we examined enrichment in a common mammalian retrotransposon, Line1. While no changes in the mRNA levels of Line1 were observed across treatment groups (Supplementary Figure 2A), 6 mA was highly enriched in a regulatory region upstream of Line1 elements in our MeDIP assay (Supplementary Figure 5). The conserved presence of 6 mA in both *Drosophila* and rat retrotransposons suggests some commonality in regulation, despite 6 mA having opposite effects on gene expression in these two species. To compare across more phylogenetically similar groups, we examined Brain expressed X-linked 4 (Bex4), an X-linked gene associated with 6mA-induced repression in mouse embryonic stem cells^14^. We observed no significant differences in Bex4 mRNA, which may suggest either differences between mice and rats or tissue-specific effects (Supplementary Figure 2A).

**Figure 6:**
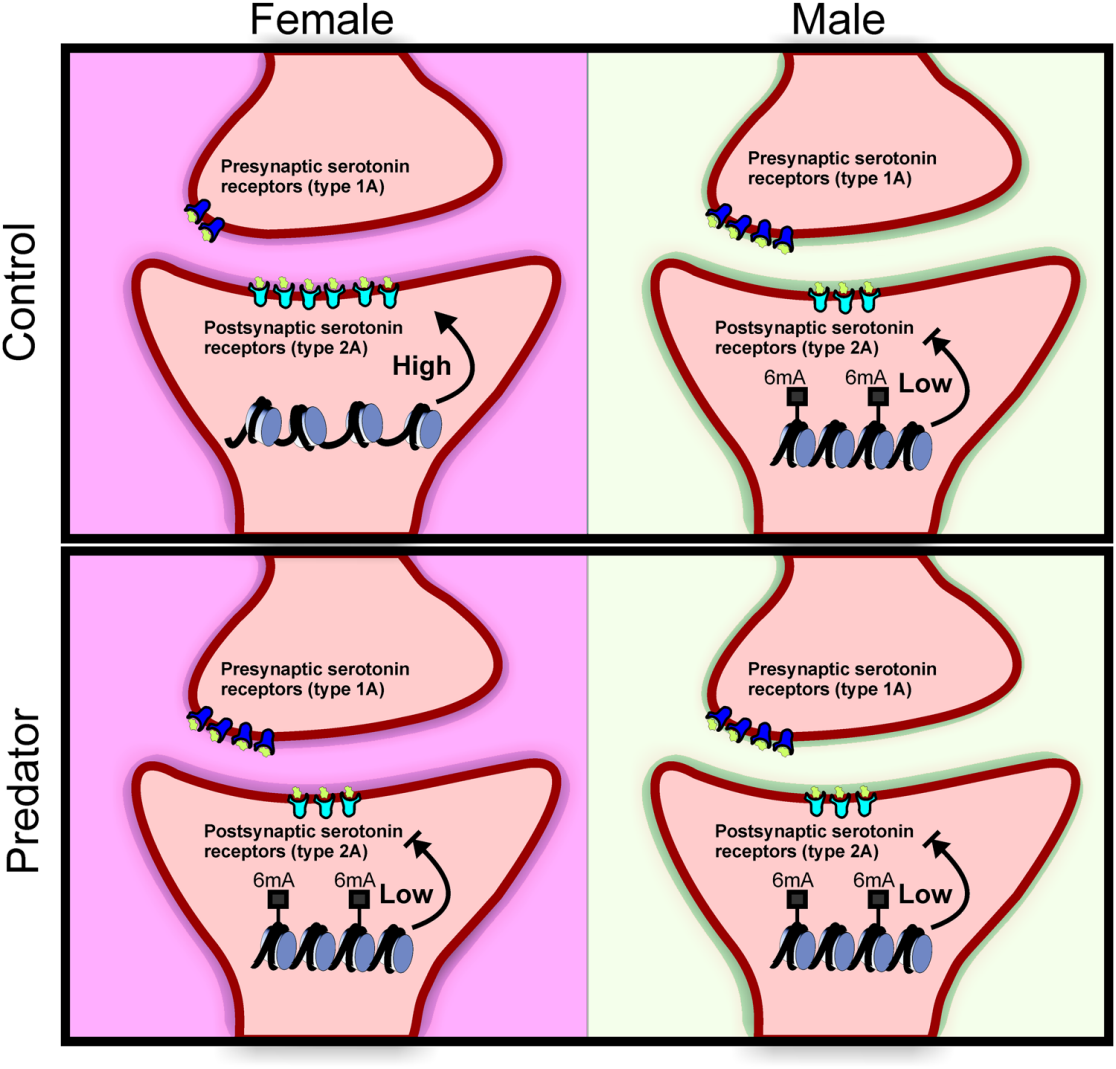
Model showing predicted effect of biological sex and stress on 5-HT signaling in the juvenile amygdala. Htr1a acts as a pre-synaptic autoreceptor, inhibiting 5-HT release into the synaptic terminal. Htr2a is expressed post-synaptically, and initiates signal transduction events in response to 5-HT signaling. Chromatin states for the Htr2a promoter are predicted to relaxed or condensed based on 6 mA status. Clockwise from top left: Control females have low levels of Htr1a and high levels of Htr2a, indicating heightened sensitivity to 5-HT signaling. Low levels of 6 mA allow for a relaxed chromatin state in the Htr2a promoter and greater access by the transcriptional machinery. Control males have both higher levels of 6 mA in the Htr2a promoter, leading to condensed chromatin and diminished transcription of Htr2a, and higher levels of Htr1a presynaptically. This would be predicted to cause blunted activation by 5-HT release. Stress males show no significant differences when compared to control males; stressed females phenocopy control and stressed males.

Finally, we performed a ChIP assay to examine chromatin state, looking at H3K9 acetylation—a well-known marker of active gene transcription^33^. We saw that males had significantly less H3K9 acetylation at Site A (Fig. 5A), consistent with the notion that 6 mA may act as a marker of gene repression in the *Htr2a* promoter as males also had more 6 mA at this location. We also looked at the transcription factor Sp1, as it is a known regulator of Htr2a expression in this region^34^; we found that females had significantly more Sp1 bound at this site (Fig. 5B). This supports a scenario in which the presence of 6 mA causes increased condensation of chromatin, reduced binding of Sp1, and ultimately reduced expression of Htr2a, summarized in Fig. 6. At present, it is unclear what regulates Htr1a mRNA expression; this will be an important avenue for future study.

To the best of our knowledge, this study reports the first evidence that 6 mA is regulated sex-specifically in the neonatal and adolescent brain by environmental perturbations such as ELS. During revision of this manuscript, another group has reported that chronic restraint stress in adult male mice leads to accumulation of 6 mA in the prefrontal cortex (PFC)^41^, which is supportive of both our findings in terms of the inverse relationship between 6 mA and gene expression and stress-induced increases in 6 mA levels. It is important to note that the existence of 6 mA in the mammalian brain remains controversial^42^, though attempts to identify this adduct in the mammalian genome were done using non-enriched HPLC analysis of whole brain, which may dilute out detectable signal. In contrast, we verified our offline-enriched HPLC results using a combination of several methods, including via PCR amplification of immunoprecipitated DNA. In sum, these data raise the intriguing possibility that 6 mA may be an important biomarker for investigating the stress-induced development of neuropathologies.

## Materials and Methods

### Animals

Untimed-pregnant Sprague Dawley female rats (~15 d pregnant) were purchased from Charles River Laboratories (Wilmington, MA) and allowed to deliver normally. Cages were checked regularly to determine the day of birth (P0). On P0, approximately 6 hours after birth, litters were culled to 10 (5 males and 5 females). Juveniles were weaned at P21 into cages of 5 with littermates into mixed-sex groups. Vaginal opening was monitored to ensure females were pre-pubertal. Weights were collected at sacrifice. For sex difference experiments, 40 unhandled animals (20 males, 20 females; representing at least six different litters to control for maternal variation) were raised to P35, and sacrificed for use in either methylated DNA or chromatin immunoprecipitation (MeDIP and ChIP) analysis. Samples were then pooled for mass spectrometry analysis. Animals were housed under standard laboratory conditions (reverse light/dark cycle of 12 h/12 h, food and water *ad libitum*). All experiments were performed in accordance with the NIH guide for care and use of laboratory animals in research and were approved by the University of Wisconsin-Madison Animal Care and Use Committee.

### Predator odor exposure

Twelve randomly assigned litters were used in this study, with at least three dams represented for each of four groups (neonatal stress, neonatal control, neonatally-stressed juvenile, and neonatally control-handled juvenile) to control for variations in maternal behavior. Pups from both stress and control groups were removed from the dam and taken to separate rooms, where they were placed in a novel (non-conditioned) odorant chamber measuring approximately 480 cm^2^. This chamber contained a perforated platform which was 23 cm × 16 cm with 192 holes, each approximately 5 mm in diameter. The platform was surrounded by 5 cm plastic walls and raised approximately 5 cm with metal legs to ensure that no physical contact with the odor occurred.

Stress group animals were exposed to cat odor (soiled cat litter and fur from ~7 different cats of mixed sexes), novel adult male rat odor (soiled bedding from rats in the facility), and ferret odor (fur and droppings from a variety of pet ferrets, purchased online as Mouse-B-Gone at http://www.mousebgone.com) on P1-3, respectively, for 5 minutes at a time (Fig. 1A); an individual monitored pup behavior throughout the 5 minute exposure period. Animals in the control group were handled in the same manner but were not exposed to any predator odor. All animals were returned to the dam in <10 minutes. A subset of 6 litters was sacrificed 30 minutes after the last odor exposure on P3. The remaining 6 litters were reared normally until behavioral testing before sacrifice on P33.

### Elevated Plus Maze (EPM)

The EPM was used to assess anxiety-like behavior, as previously described^43^. Parameters quantified were entries into as well as time spent in the open arm, head dips, and stretch attenuated postures. An entry was counted when all four paws crossed into any arm of the maze. The experiment was performed under dim red light beginning 2 hours after the dark phase of the light cycle began. Animals were not repeat-tested in the task. Each animal was video recorded and behavior was analyzed and scored using The Observer software (Noldus Information Technologies, Leesburg, VA) by a trained observer blind to all treatments. One stressed male behavioral outlier was removed from all analyses.

### Tissue collection

Both neonatal and juvenile rats were sacrificed via rapid decapitation. The amygdala was dissected as previously described^43^ and immediately snap frozen in isopentane on dry ice. Tissue samples were first randomized and then homogenized; total RNA and DNA were collected using an AllPrep DNA/RNA Mini Kit (Qiagen, Germantown, MD; catalog #80204). The P3 amygdala set contained 6 control females, 6 stress females, 6 control males, and 6 stress males; 3 distinct litters were represented in each group to minimize any effects of variation in maternal care. The P33 amygdala set contained an n = 10 animals per group (except the stressed male group, which contained an n of 8 animals), with at least three litters represented in each as before.

### Quantitative PCR Analysis

RNA conversion to cDNA for RT-qPCR analysis was performed as previously described^43^. Following amplification, a dissociation melt curve and DNA gel analysis was performed to ensure specificity of the primers. cDNA levels were normalized to three averaged housekeeping genes—Ywhaz, Hprt, and Rpl13a—using a modified version of the ΔΔCT method to determine relative quantitation of gene expression. mRNA primer sequences are listed in Supplementary Table 1.

### Enzyme-based Methylation Analysis

5 mC DNA methylation was assessed using an adapted version of the methylation sensitive restriction enzyme (MSRE) assay^44^, as previously described^39,43^. Primers were designed such that some combination of HpaII (New England Biolabs, Ipswich, MA; catalog #R0171), HhaI (New England Biolabs; catalog #R0139), or Hpy188III (New England Biolabs; catalog #R0622) cut sites were inside or outside of the primer sequence. 6 mA DNA methylation was determined by methylation dependent restriction enzyme (MDRE) analysis. Briefly, 240 ng of DNA from each rat was divided equally into two tubes and digested with either DpnI (Life Technologies, Carlsbad, CA; catalog #5242-019) or BstUI (Life Technologies; catalog #ER0922) in the same buffer conditions at 37 °C for 3 hours. Enzymes were subsequently inactivated by heating to 70 °C for 15 min. A no-DNA control was added to ensure purity of the restriction enzyme reaction. For MDRE, primers were designed such that DpnI cut sites, but not BstUI cut sites, were contained within the region of interest. After cutting, qPCR was performed as described above to quantify relative amounts of DNA methylation. Genomic DNA primer sequences are listed in Supplementary Table 2.

### Enzymatic Digestion of Genomic DNA for Mass Spectrometry (MS)

All chemicals and enzymes, unless otherwise specified, were purchased from Sigma-Aldrich (St. Louis, MO) and New England Biolabs. Erythro-9-(2-hydroxy-3-nonyl) adenine (EHNA) hydrochloride was purchased from Tocris Bioscience (Ellisville, MO). *D*_3_ *-N* ^6^-*methyl-deoxyadenosine* were synthesized using established procedures^45^. DNA from between 4 and 8 individual animals’ amygdalae was pooled for each biological replicate to obtain ~20–28μg of DNA; there were 4 biological replicates for each group, male and female, processed separately. For the enzymatic digestion of DNA, nuclease P1 (0.1 U/μg DNA), phosphodiesterase 2 (0.00025 U/μg DNA) and erythro-9-(2-hydroxy-3-nonyl)adenine (1 mM in final concentration) were added to DNA in a solution containing 30 mM sodium acetate (pH 5.6) and 1.0 mM zinc acetate. The digestion mixture was incubated at 37 °C for 48 h and continued for 2 h after the addition of alkaline phosphatase (0.05 U/μg DNA), phosphodiesterase 1 (0.0005 U/μg DNA) and 0.5 M Tris–HCl (pH 8.9). Enzymes were then removed via chloroform extraction and the aqueous layer was dried by Speed-vac. DNA samples were then reconstituted in deionized distilled water and their concentrations measured using UV-absorption spectrophotometry. To ~20–28 μg of digested nucleosides were then added stable-isotope labeled 6 mA [formally, 6-mdA] (10 fmol) and the spiked sample was subjected to off-line HPLC enrichment. For the LC-MS/MS analysis of 5 mC [formally, 5-mdC], approximately 50 ng of the digested mixture was used.

### HPLC Enrichment and LC-MS/MS Analysis of 5 mC and 6 mA

A 4.6 × 250 mm GraceSmart RP18 column (5 μm in particle size, Grace Davison, Deerfield, IL) was used to enrich 6 mA from the enzymatic digestion products of DNA. The flow rate was 0.5 mL/min, and the mobile phases were 10 mM ammonium formate (solution A) and methanol (solution B). Gradients of 15 min at 2–15% B, 15 min at 15–35% B, 10 min at 35–60% B, and 5 min at 60–80% B were employed. The HPLC fractions eluting at 35–38 min were pooled for 6 mA enrichment (Supplementary Figure 6). The collected fractions were dried under Speed-vac, redissolved in H_2_O, and subjected to LC-MS/MS analysis. A 2.1 × 50 mm Acquity UPLC BEH Amide column (1.7 μm particle size, Waters, Milford, MA) and a Prominence UFLC-XR pump (Shimadzu, Columbia, MD) were used, with a flow rate of 400 μL/ min. A solution of 0.1% (v/v) formic acid in deionized distilled water (solution A) and a solution of 0.1% (v/v) formic acid in methanol (solution B) were employed as mobile phases. An isocratic gradient of 2% B was implemented for 10 min. The effluent from the LC column was directed to a QTRAP 5500 mass spectrometer (SCIEX, Framingham, MA). The instrument was operated in multiple reaction monitoring (MRM) mode, and the MRM transition measured for 5 mC was *m/z* 242 → 126. LC-MS/MS analysis of unlabeled and labeled 6 mA was performed with MRM transitions of *m/z* 266 →150 and 269 →153, respectively.

### Methylated DNA Immunoprecipitation (MeDIP)

Genomic DNA, isolated from non-handled P35 males and females (n = 6 per group), was fragmented using probe-based sonication. Shearing was confirmed via DNA gel analysis to be between 300–1000 bp. 800 ng of sonicated DNA was incubated at 4 °C overnight with either 6 mA antibody (Millipore, Darmstadt, Germany; catalog #ABE572) or 5 mC antibody (Millipore; catalog #MABE146). An IgG negative control reaction was included to test for non-specific DNA-binding. The antibody-DNA sample was then incubated with a Dynabead immunoprecipitation kit (Life Technologies; catalog #10007D) for 2 hours at 4 °C and the manufacturer’s protocol followed. qPCR analysis was performed to quantify relative amounts of methylated DNA enrichment; immunoprecipitated (IP) samples were normalized to input.

### Chromatin Immunoprecipitation (ChIP)

ChIP was performed on bilateral amygdalae sections collected as described above from non-handled P35 males and females (n = 6 per group). Snap-frozen tissue (~37 mg) was fixed in 1% formaldehyde for 15 min at room temperature with gentle shaking. The reaction was quenched with 160 uL of 1.25 M glycine for 5 min at room temp with gentle shaking. Samples were washed 2x with PBS and cOmplete protease inhibitor (CPI) tablets (Roche, Basel, Switzerland; catalog #04693116001) then resuspended in 400 μL RIPA lysis buffer + CPI tablets. Samples were homogenized with a pestle then lightly sonicated via probe-based sonication to fully lyse cells and the nuclear envelope. Chromatin shearing was obtained using 1000 gel units of Micrococcal Nuclease (MNase) (New England Biolabs; catalog #M0247S); samples were incubated with Proteinase K to ensure efficient shearing of DNA (between 300 bp–900 bp). The MNase reaction was stopped with 1.25 μmol EGTA. Debris was removed by centrifugation at 15,000 g for 10 m at 4 °C; 100 μL of each lysate was diluted in 200 μL IP buffer (PBS + 0.05% Triton X-100) and incubated overnight with 1 μg of antibody. Antibodies used were H3K9Ac (Upstate, Lake Placid, NY; catalog #06-942) and Sp1 (Santa Cruz, Dallas, TX; catalog #sc-59x). After overnight antibody incubation, 25 μL of magnetic beads (Life Technologies; catalog #10004D) was added and mixed for 2 h at 4 °C with gentle rocking. Samples were washed sequentially with 500 μL Low Salt, 500 μL High Salt, and 500 μL TE Buffers, then resuspended in Digestion Buffer (50 mM Tris, 10 mM EDTA, 0.5% SDS, pH 8.0) + Proteinase K for 2 h at 62 °C. DNA was isolated via column clean-up. PCR was then performed on samples as described above.

### Statistical Analysis

Statistics were run using SigmaPlot 11. Behavioral data, mRNA PCR data, and MDRE/MSRE data were analyzed using two-way ANOVA and Tukey post-hoc; MeDIP and ChIP data were analyzed using t-tests. Potential outliers were screened for using the Grubbs test for outliers. All reported measures are listed as mean ± SEM. Significance was defined as a *p* value of <0.05.

## Electronic supplementary material

SUPPLEMENTARY FIGURES
