# Supplementary material for: N6-methyladenine is an epigenetic marker of mammalian early life stress
Source: Sci Rep. 2017 Dec 22;7:18078. doi: 10.1038/s41598-017-18414-7 (PMC5741724; doi:10.1038/s41598-017-18414-7)
Supplement: Supplementary file 1 — SUPPLEMENTARY FIGURES [file 41598_2017_18414_MOESM1_ESM.pdf]

N^6-methyladenine is an epigenetic marker of mammalian early life stress

Stacey L. Kigar[1], Liza Chang[2], Candace R. Guerrero[3], Jacqueline R. Sehring[2], Amelia Cuarenta[2], Laurie L. Parker[3], Vaishali P. Bakshi[4], Anthony P. Auger[2,5]

[1.] Molecular and Cellular Pharmacology Training Program, [2.] Department of Psychology, University of Wisconsin-Madison, [3.] Department of Biochemistry, Molecular Biology, and Biophysics, University of Minnesota, Minneapolis, MN 55455, [4.] Department of Psychiatry, University of Wisconsin-Madison, [5.] Neuroscience Training Program, University of Wisconsin-Madison, Madison, WI 53706.

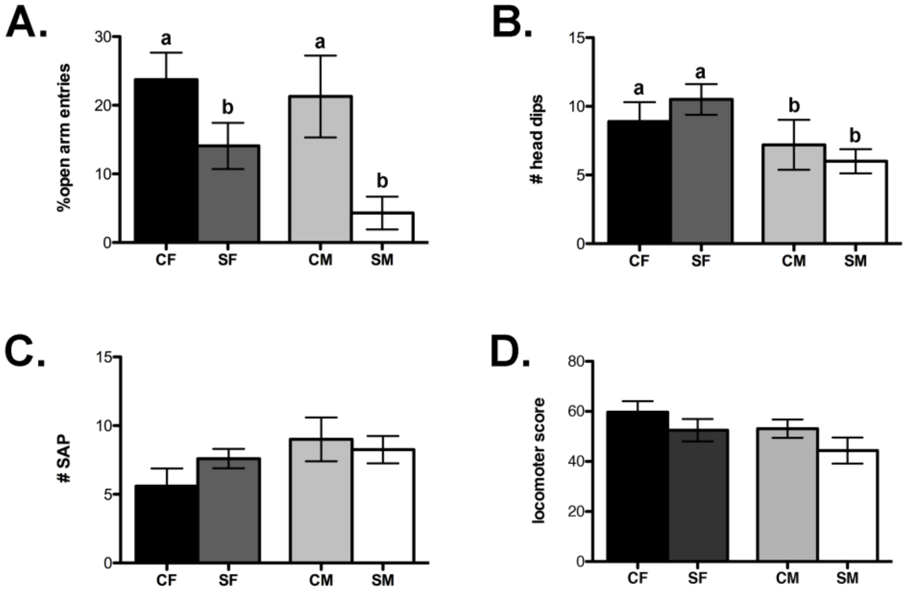

Supplementary Figure 1: Anxiety-like behavior testing in the elevated plus maze (EPM). *(A)* A significant treatment effect on number of crosses into the open arm as a percentage of all crosses was observed *(B)* A significant sex difference in the number of head dips was observed (two-way ANOVA; $F_{(1,34)} = 4.843$, $p = 0.035$). *(C)* There were no effects on the number of times animals took the stretch attend posture. *(D)* We assessed general activity in these rats by combining the number of total crosses in the EPM, with the number of approaches to an object in a separate behavioral task; there was no significant effect of stress or sex on movement.

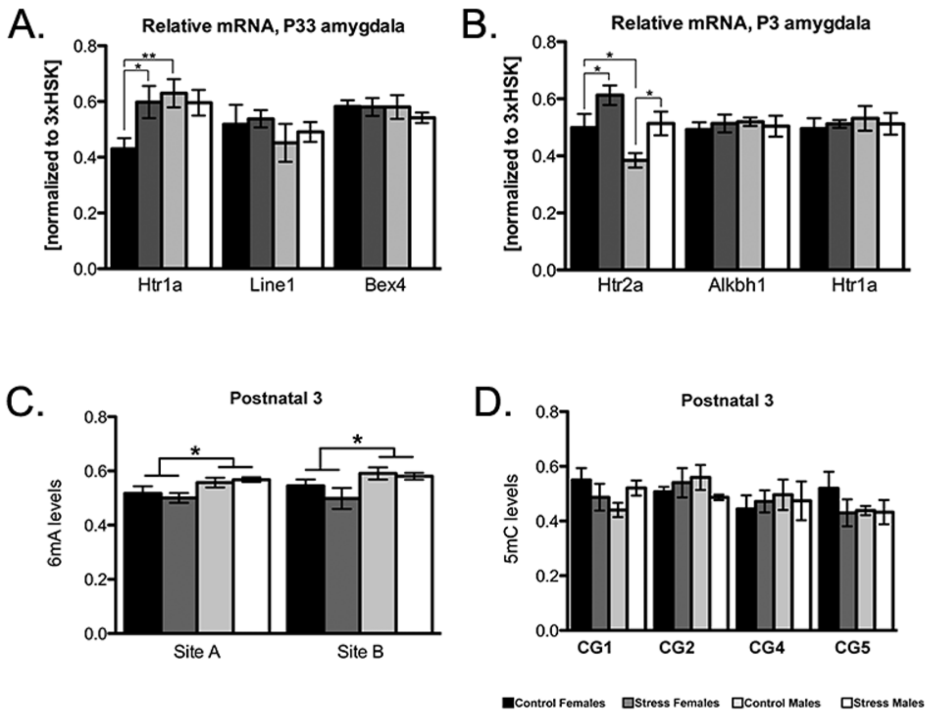

Supplementary Figure 2: RT-qPCR data showing the effects of neonatal POE and biological sex on gene expression and DNA methylation in the amygdala. *(A)* Significant difference in the mRNA expression of Htr1a that are opposite that of Htr2a in the juvenile amygdala. There were no group differences in mRNA expression of the retrotransposon Line1 or the X-linked gene Bex4. *(B)* mRNA expression in the P3 amygdala shows sex and treatment effects on Htr2a, but not Htr1a or Alkbh1. There were no changes in Line1 or Bex4 (data not shown). *(C)* Sex differences in 6mA abundance are present at both sites in the *Htr2a* promoter neonatally. *(D)* No differences in 5mC were observed (levels of methylation were below the limit of detection at CG site 3). Values shown as mean ± SEM, $**p < 0.01$, $*p < 0.05$.

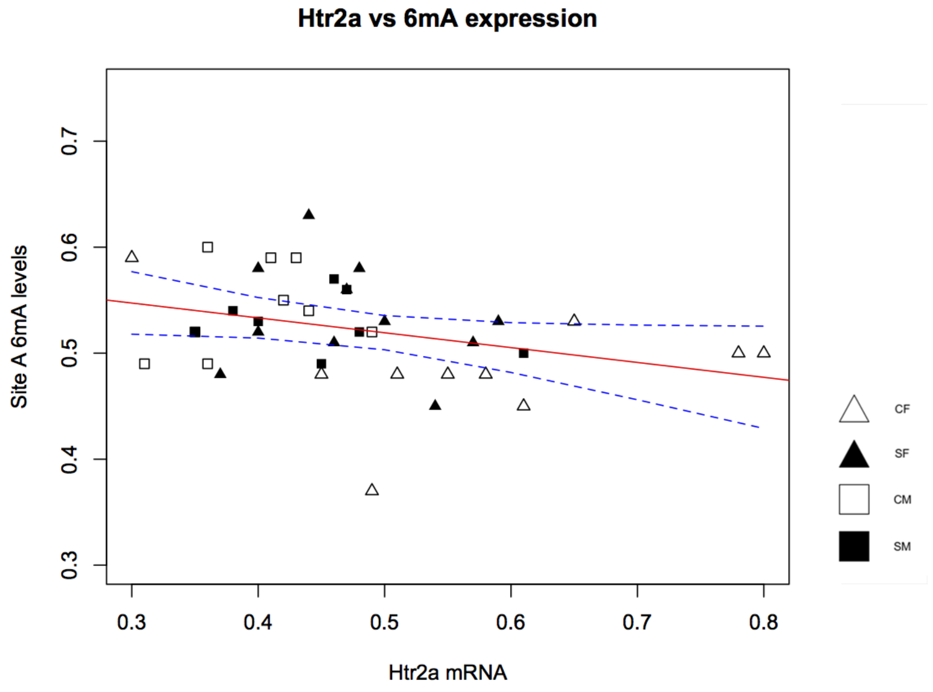

Supplementary Figure 3: Regression analysis showing trending correlation between relative levels of 6mA abundance and Htr2a mRNA expression levels in the P33 amygdala. $p = 0.0509$, $F_{1,36} = 4.079$.

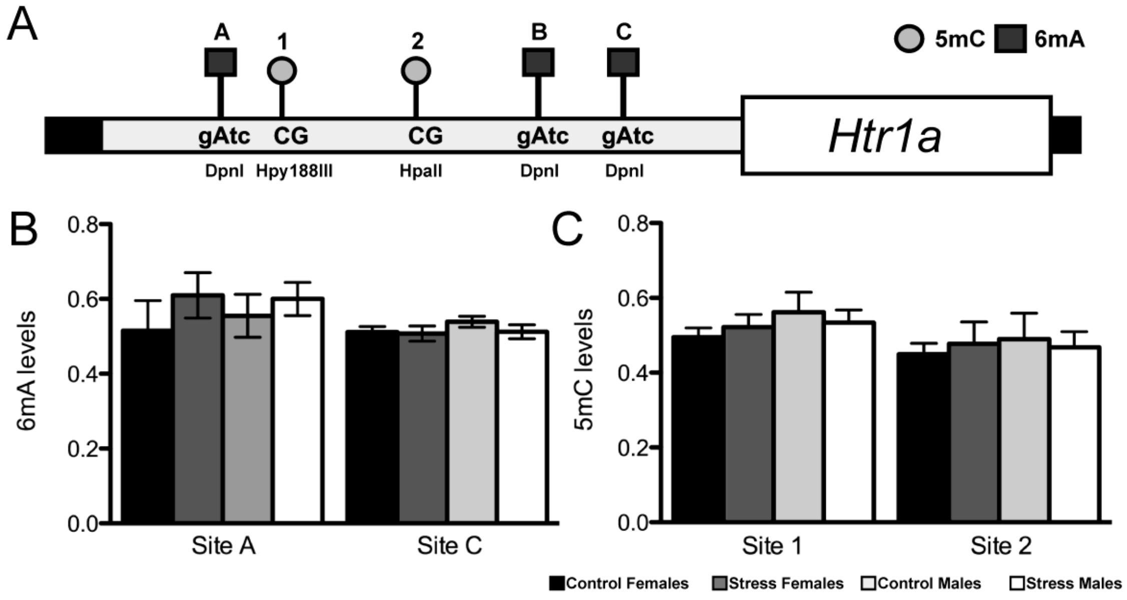

Supplementary Figure 4: *(A)* Schematic of *Htr1a* gene promoter, showing individual methylation sites analyzed. *(B)* MDRE analysis shows no differences in relative 6mA methylation at two distinct GATC sites in the *Htr1a* promoter. Note: no methylation was detected at Site B. *(C)* MSRE analysis shows no differences in 5mC methylation at two distinct CG sites. Sites A, C, 1, and 2 were explored using MeDIP analysis; there were no significant differences (data not shown). Values shown as mean ± SEM.

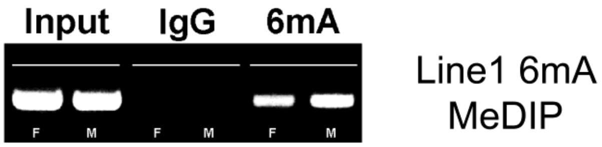

Supplementary Figure 5: DNA gel for 6mA MeDIP of Line1 showing robust enrichment, as well as input and IgG controls.

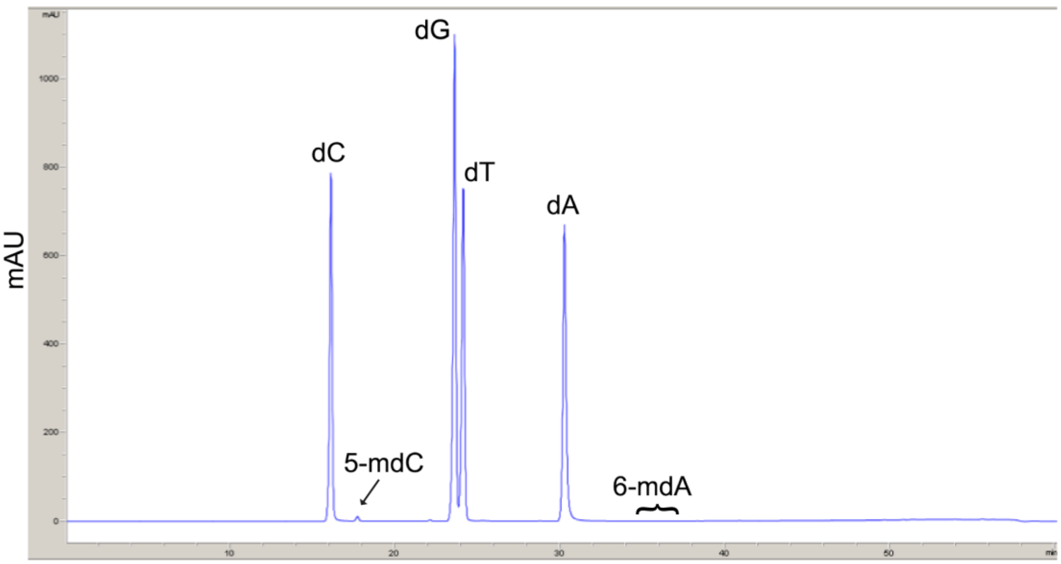

Supplementary Figure 6: Representative enrichment chromatogram of enzymatically digested DNA from P35 amygdala showing separation of nucleoside peaks and their relative abundance.

Primer	Accession	Forward	Reverse
**Ywhaz**	NM_013011	TTGAGCAGAAGACGGAAGGT	GAAGCATTGGGGATCAAGAA
**Hprt**	NM_012583	GCAGACTTTGCTTTCCTTGG	CCGCTGTCTTTTAGGCTTTG
**Rpl13a**	NM_173340	AGCAGCTCTTGAGGCTAAGG	GGGTTCACACCAAGAGTCCA
**Alkbh1**	NM_001108718	GCGGAGACCCCGAAGTTTAC	TGGCGACTTGCTCTTACTGT
**Htr2a**	NM_017254	AACGGTCCATCCACAGAG	AACAGGAAGAACACGATGC
**Htr1a**	NM_012585	CCGCACGCTTCCGAATCC	TGTCCGTTCAGGCTCTTCTTG
**Bex4**	NM_001037554	GAAAACAAGAAGCCTGGGGG	GCGTGTAAGGCCTCATCTGT

Supplementary Table 1: mRNA Primer sequences and Pubmed accession numbers for RT-qPCR.

Promoter	Location:	Forward	Reverse
**Htr2a**	Site #1 – Hpy188III	AAAGTAAGCTAGTTGCGAGATGTA	GTCAGGAAGTCTGTGGCAATAA
**Htr2a**	Site A - DpnI	CCTGACTCCTCTGAACGTGT	GTCCTGGGATCTAGGGGCAT
**Htr2a**	Site #2 - HpaII	CCTAGGCTGTCTCCCCATTC	TGCCTCCTTCCTCAGACCTC
**Htr2a**	Site #3 - HhaI	TGCGGCTCTTTTGTGTGACT	AGCAGCCCAGGAACTTACAT
**Htr2a**	Site #4 - HpaII	TGCGGCTCTTTTGTGTGACT	AGCAGCCCAGGAACTTACAT
**Htr2a**	Site B - DpnI	CTCCCTCCTCGTTTGGATCT	GCTGTAAGTTCTCACGGAAGC
**Htr2a**	Site #5 - HpaII	CTCCCTCCTCGTTTGGATCT	GCTGTAAGTTCTCACGGAAGC
**Htr1a**	Site A - DpnI	AGGAGGCGGGGTTTAATCTG	CCCCCACCACCATCTAACAC
**Htr1a**	Site #1 – Hpy188III	AGGAGGCGGGGTTTAATCTG	CCCCCACCACCATCTAACAC
**Htr1a**	Site #2 - HpaII	GAGAGAAGCAACCAGGAGATG	GGATTCTCCCGCCTAACAAA
**Htr1a**	Site B - DpnI	TGAGTGCTCTTCTCAGATGCC	TTTTCTGGGGAGTTTCAGAGGG
**Htr1a**	Site C - DpnI	TCCCTCTGAAACTCCCCAGAA	AGGTCACGTCGGAGATGCTA
**Line1**	----	GAAAGCACCAAATGCCACTGG	GTAGTCTGCTATCGGGCGTT

Supplementary Table 2: Genomic DNA promoter primer sequences for RT-qPCR. *Htr2a* accession number: L31546. *Htr1a* accession number: AF217200.
